# MMG-Based Knee Dynamic Extension Force Estimation Using Cross-Talk and IGWO-LSTM

**DOI:** 10.3390/bioengineering11050470

**Published:** 2024-05-09

**Authors:** Zebin Li, Lifu Gao, Gang Zhang, Wei Lu, Daqing Wang, Jinzhong Zhang, Huibin Cao

**Affiliations:** 1 Anhui Undergrowth Crop Intelligent Equipment Engineering Research Center, West Anhui University, Lu’an 237012, China; zhangjinzhongz@126.com; 2 Institute of Intelligent Machines, Hefei Institutes of Physical Science, Chinese Academy of Sciences, Hefei 230031, China; lifugao@iim.ac.cn (L.G.); dqwang@mail.ustc.edu.cn (D.W.); hbcao@iim.ac.cn (H.C.); 3 Department of Science Island, University of Science and Technology of China, Hefei 230031, China; 4 School of Management, Fujian University of Technology, Fuzhou 350118, China; lw9296@mail.ustc.edu.cn

**Keywords:** knee dynamic extension force estimation, mechanomyography, crosstalk, grey relational analysis, long short-term memory network, improved grey wolf algorithm

## Abstract

Mechanomyography (MMG) is an important muscle physiological activity signal that can reflect the amount of motor units recruited as well as the contraction frequency. As a result, MMG can be utilized to estimate the force produced by skeletal muscle. However, cross-talk and time-series correlation severely affect MMG signal recognition in the real world. These restrict the accuracy of dynamic muscle force estimation and their interaction ability in wearable devices. To address these issues, a hypothesis that the accuracy of knee dynamic extension force estimation can be improved by using MMG signals from a single muscle with less cross-talk is first proposed. The hypothesis is then confirmed using the estimation results from different muscle signal feature combinations. Finally, a novel model (improved grey wolf optimizer optimized long short-term memory networks, i.e., IGWO-LSTM) is proposed for further improving the performance of knee dynamic extension force estimation. The experimental results demonstrate that MMG signals from a single muscle with less cross-talk have a superior ability to estimate dynamic knee extension force. In addition, the proposed IGWO-LSTM provides the best performance metrics in comparison to other state-of-the-art models. Our research is expected to not only improve the understanding of the mechanisms of quadriceps contraction but also enhance the flexibility and interaction capabilities of future rehabilitation and assistive devices.

## 1. Introduction

Knee movement is an essential part of lower limb movement, and knee force produced by skeletal muscle plays a critical role in completing lower limb movements and interacting with the external environment. In recent years, rehabilitation and assistive devices have achieved a wide range of application prospects in areas such as rehabilitation medicine, military operations, and disaster relief. To improve the naturalness and flexibility of device movement, these devices need to know the human movement intention before human movement [1]. Therefore, it is vital to supply effective intelligent control information, which will directly affect the comfort and efficient interaction of these devices [2].

Over the past decades, a lot of work has been performed by many experts using muscle activity to predict joint angle [3], acceleration [4], torque [5], muscle force [6,7,8,9], fatigue effect [10], etc. In particular, estimating muscle force from muscle activity in these investigations is a challenging task, which has many potential applications such as diagnosis of muscle dysfunction, rehabilitation training, prosthetic assistive devices, etc. [11,12].

In general, direct measurement of muscle force requires a complicated and costly acquisition device, limiting not only portability but also real-time performance. Therefore, many researchers have investigated indirect ways of assessing muscle force utilizing human surface bio-signals, such as surface electromyography signals (sEMG) and MMG.

sEMG can represent the number and firing rates of active motor units, which are closely related to muscle activity during muscle contraction, making it a reliable mainstream approach for estimating skeletal muscle force/torque [13,14]. Although sEMG contains abundant physiological motion information and reflects human motor intention, and has been proven to be closely related to the corresponding muscle activity [15], it is severely limited in related domains due to relatively small signal amplitude, interferences from other electrical equipment, skin impedance changes, etc.

MMG, which is pressure waves created by the activation of muscle fibers and their dimensional changes during muscle contraction [5], and represents the muscles’ mechanical output, is comparable to sEMG. Thus, MMG can also provide important information on muscle activity and motor unit recruitment patterns [16], which can be detected on the skin surface above the target muscle by accelerometers, piezoelectric microphones, etc. [1,4,13,17]. Even though MMG is influenced by many factors such as muscle morphology and the physical environment [18], it has significant advantages over sEMG with no skin preparation, negligible skin impedance, no need for precise test positioning, and less electronic noise interference [13]. Notably, MMG has been applied to human motion recognition and kinetic parameter estimation [1,4,5,6,13,18,19,20,21]. However, it is still a challenging issue to establish direct relationships between MMG signals and interactive forces due to the complexity and variety of muscle motor unit (MU) recruitment. One of the problems that cannot be ignored is cross-talk during muscle contraction, which is a critical issue that prevents the clinical application of MMG [22].

Cross-talk in MMG signals refers to the contamination of the signal from the muscle of interest by the signal from another muscle or muscle group in very close proximity [23]. Some scholars have conducted considerable study on cross-talk, indicating that cross-talk seems to be related to many factors, including anthropometric parameters, the direction of the collected signal, the proximity of the muscles, etc. Talib et al. [24] employed cross-correlation coefficients (CCC) to quantify the magnitude of cross-talk between elbow flexor muscle pairs at 80% of maximum voluntary isometric contraction (MVIC) and found the root mean square (RMS) and cross-talk seemed to be unrelated to anthropometric parameters. Beck et al. [25] found a strong correlation between MMG signals detected in the vertical and horizontal axes, implying that detecting MMG signals in the multi-axis direction may be unnecessary. Among these factors, cross-talk from adjacent muscles is the most direct. Mohamad Ismail et al. [10] investigated cross-talk in MMG signals from forearm extensors and flexors before and after fatigue, and found that cross-talk is consistently higher in the extensors than the flexors and that cross-talk values increase as the distance between two adjacent muscles decreases. In addition, related evidence indicates that cross-talk in forearm muscle MMG signals [26] is greater than in quadriceps MMG signals [27], possibly due to the closer distance between forearm muscles than quadriceps muscles.

In specific rehabilitation and assistive device control, muscle force often needs to be identified. The above reports also further indicate that all MMG signals obtained from the muscle surface are affected by cross-talk. Unfortunately, the participation of muscles in joint movements is still not well understood. Similarly, the effect of cross-talk in muscle signals on muscle force estimation has been also consistently overlooked. It may also be an important reason of poor accuracy of the estimation of the relevant muscle force. For example, Youn et al. [28] did not take cross-talk into account in estimating elbow flexion force, and obtained results with the normalized root mean square error (NRMSE) of not less than 0.130 and the correlation coefficient (R) of not more than 0.904; similarly, in another of their studies on elbow flexion force estimation, they also obtained the best results of NRMSE of not less than 0.1 and R of not more than 0.93 [18]. Furthermore, a similar occurrence was observed in both MMG-based and sEMG-based related muscle force estimation studies [6,8,9,15,29]. Nevertheless, there have been no reports so far on how to identify a muscle with less cross-talk for muscle force estimation.

When MMG signals are employed for muscle force estimation, apart from the consideration of cross-talk, a significant technical difficulty is the development of an appropriate muscle force estimation model. In the previous stage, we constructed knee static extension force estimation models using support vector regression (SVR) [30,31] and achieved high accuracy. However, when applying these models to knee dynamic extension force estimation, the results were not satisfactory. Recent related works [7,9,28,32] have also reported unsatisfactory results for dynamic muscle force estimation using traditional machine learning methods. Consequently, we would like to continue to investigate suitable models for dynamic muscle force estimation.

Among the data-driven approaches, the long short-term memory (LSTM) network is well suited for non-linear and time-series data [33], which not only effectively alleviates the problem of gradient explosion and gradient disappearance in a recurrent neural network (RNN), but also solves the long-time dependence problem of RNN [34]. Dao [35] built a lower limb muscle strength prediction model using a developed LSTM network and achieved an accuracy of relative root mean square error (RMSE) deviation of less than 5% and Pearson correlation coefficients of greater than 95% in a real-world database, respectively. Chen et al. [34] proposed an LSTM-based continuous estimation model for estimating three-dimensional motion of the upper shoulder elbow joint (touch task and composite task) and achieved high estimation accuracies, with the coefficient of determination (R^2^) of 0.9171 for the touch task and R^2^ of 0.8109 for the composite task. In addition, compared to the multi-layer perceptron (MLP) model, the proposed model’s root mean square error (RSME) was reduced by 13.57%.

In an LSTM network, hyper-parameters such as the learning rate and the number of hidden layer neurons affect the prediction accuracy in addition to determining the training effect and training speed of the network. Recent investigations have demonstrated that the LSTM can achieve the same performance as a more complex structured LSTM model if its hyper-parameters are carefully tuned or optimized [36]. When Rashid et al. [37] compared the grey wolf optimizer (GWO) to other optimization algorithms, they noticed that the LSTM optimized by GWO provided the best performance. However, GWO is prone to falling into local optima in the later stages of the optimization search process and has a slow convergence rate in the early stages [38]. Therefore, to better balance the algorithm’s global and local search capabilities, this investigation employs the previously developed IGWO to construct an IGWO-LSTM estimation model.

Herein, based on the characteristics of the knee extensor muscles of the lower limb, this article chooses to use accelerometers placed on the knee extensor muscles to obtain the MMG signals. Then, muscle signals with low cross-talk can be identified by cross-talk analysis, which can be used as signals for dynamic knee extension force estimation to improve the estimation accuracy. Additionally, in order to further improve the accuracy of the dynamic extension force estimation, an IGWO-LSTM-based dynamic extension force estimation model is proposed. The remaining sections are organized as follows. Section 2 summarizes in detail the subject profile, experimental equipment and procedures, signal processing method, data cross-talk analysis method, model design process, and model evaluation indicators. Section 3 presents cross-talk between different muscle pairs, the estimation results for different combinations of muscle signals, and comparison of different models for the knee dynamic extension force estimation. Section 4 discusses experimental results, advantages, and limitations of the current work. Finally, Section 5 presents the conclusions and the potential for its application, as well as future issues that need to be addressed.

## 2. Materials and Methods

### 2.1. Experimental Devices and Procedures

Six healthy subjects, five males and one female (mean ± SD age = 19.83 ± 1.47 years; body weight = 75.33 ± 22.02 kg; height = 1.78 ± 0.08 m), with no history of neuromuscular injury, volunteered to participate in the experiment. The relatively small age range of subjects was intended to minimize the potential effect of age, which has been reported to be associated with age-related factors in MMG signals during force generation [39]. The experiment was approved by the Institutional Review Committee of Hefei Institute of Physical Science, Chinese Academy of Sciences, and all participants completed a health history questionnaire and signed an informed consent form before testing.

**Figure 1:**
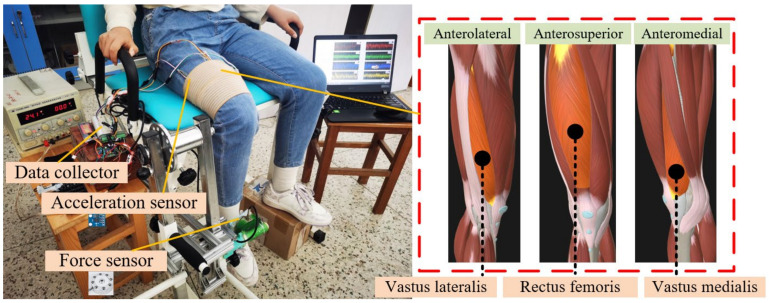
Experimental setup and quadriceps anatomy.

The experimental setup and quadriceps anatomy are illustrated in Figure 1. The signal acquisition equipment and sensor placement referenced pre-work [31]. The subjects were asked to sit comfortably in a test chair with their right leg fixed and bent at a 90° angle.

Each subject was required to visit the laboratory twice, once for the familiarization session and once for the signal acquisition session. During the familiarization session, subjects were not only familiarized with the experimental equipment but also provided with training in isometric contraction experiments, while maximum voluntary isometric contraction (MVIC) was collected, providing strong verbal encouragement during this session. The highest value of the measured force from three maximal muscle actions was recorded as a contraction level of 100% MVIC. Additionally, a percentage of MVIC (%MVIC) can reduce the influence of the maximum knee extension force value of different subjects. In the signal acquisition session, to implement knee dynamic extension force estimation during isometric contraction, firstly, static knee extension during voluntary isometric contraction (SVIC) was carried out in 10% increments from 20% to 60% MIVC; finally, knee dynamic extension during voluntary isometric contraction (DVIC) was carried out in the range of 20–60% MIVC. MMG signals obtained from three muscles (vastus medialis (VM), vastus lateralis (VL), and rectus femoris (RF)) and knee dynamic extension force obtained from the lower calf were collected simultaneously during each voluntary muscle contraction action. Each knee dynamic extension task lasted 1 min. During the experiment, subjects were not allowed to talk or move their bodies to avoid sudden variations in knee extension force and were given a two-minute rest between two adjacent voluntary muscle contraction actions to avoid muscle fatigue.

### 2.2. Signal Processing

**Figure 2:**
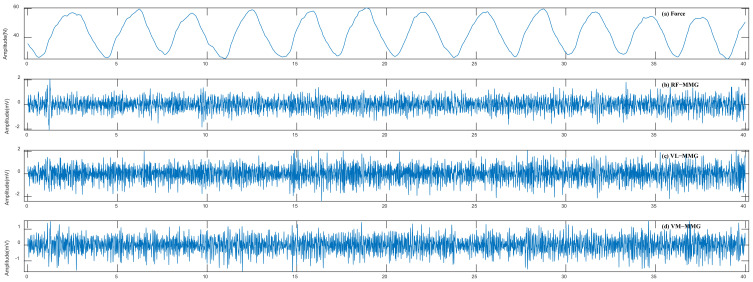
Denoised signals. (**a**) Knee extension force signals. (**b**) MMG signals from RF. (**c**) MMG signals from VL. (**d**) MMG signals from VM.

MMG signals can be obtained by accelerometers on the skin surface above the muscle of interest. Such sensors, as elaborated by Harrison, can measure vibration signals created by the contraction of muscle, but they also pick up disturbances such as the movement of the limb as it accelerates, decelerates, is lifted, or lowered [17]. To overcome these issues, we employed the previously designed noise suppression and artifacts removal method [40] to filter out these interferences. Meanwhile, for the knee extension force signals, we designed a 5 Hz third-order Butterworth low-pass filter to filter out high-frequency noise. After signal processing, we obtained a more stable 40 ms signal sequence as experimental data by deleting the 10 ms signal sequence preceding and following the original sequence. Figure 2 shows the filter effect on the original MMG signals and the knee extension force signals during DVIC.

MMG signals can reflect the mechanical properties of the muscle fibers recruited during voluntary contractions and usually have non-stationary and non-linear properties. Therefore, it is necessary to consider time domain features, frequency domain features, time–frequency domain features, and non-linear features to describe MMG signals to adequately reflect muscle activity. To this end, we extracted 25 features from each segment, and a total of 75 features from the three channels [31]. Not all of these features are highly correlated with knee dynamic extension force. Therefore, in this paper, the grey relational analysis (GRA) method from the previous work was used for feature screening to obtain valid features that are highly correlated with knee dynamic extension force [31]. Valid features screened from different subjects using the GRA method are not the same due to individual differences. In addition, for real-time estimation of knee extension force during feature extraction, we used a window length of 250 data points (250 ms) and a sliding overlap length of 50 data points (50 ms).

### 2.3. Data Cross-Talk Analysis

To evaluate the cross-talk of the three muscles (RF, VL, and VM), the denoised MMG signals were used for the cross-talk analysis in this paper. The cross-talk in the MMG signals from the two associated muscles was quantified using cross-correlation coefficients (CCC) which were calculated using Equation (1) [10,23,41]:

(1) $$R_{MMG1,MMG2}(\tau )=\frac{1}{a\times b\times \omega (\tau )}\sum_{n=0}^{N-1}\;{MMG1}_{t}(n){MMG2}_{t}(n+\tau );1-N<\tau <M$$

where ${MMG1}_{t}$ and ${MMG2}_{t}$ are MMG signals from two associated muscles, *N* and *M* are the lengths of ${MMG1}_{t}$ and ${MMG2}_{t}$, t represents the time lag between MMG signals taken from 1 − *N* to *M*, and a, b, and ω are given by Equations (2), (3), and (4), respectively.

(2) $$a=\sqrt{\sum_{n=0}^{N-1}\;{MMG1}_{t}^{2}(n)}$$

(3) $$b=\sqrt{\sum_{n=0}^{N-1}\;\;\;{MMG2}_{t}^{2}(n)}$$

(4) $$\omega (\tau )=\left\{\begin{array}{c} \frac{max(M,N)+\tau }{max(M,N)},-N<\tau <0\\ 1\;,\;\tau =0\\ \frac{max(M,N)-\tau }{max(M,N)},0<\tau <M \end{array}\right.$$

The peak cross-correlation coefficients (PCCC) in three muscle pairs, namely, RF and VL (MP1), RF and VM (MP2), and VL and VM (MP3), were employed to quantify cross-talk. The cross-correlation coefficients range from 0 to 1, where 0 indicates no common signal between the two muscles and 1 indicates 100% common signal. In cross-correlation analysis, a CCC less than 0.30 usually indicates a low correlation between signals and is considered a specific, isolated signal without cross-talk, while a CCC between 0.30 and 0.70 indicates a moderate correlation between signals and a CCC greater than 0.70 indicates a strong correlation between signals [22].

### 2.4. Estimation Model

#### 2.4.1. BPNN Model

BPNN is a multi-layer feed-forward neural network trained by a backpropagation algorithm based on gradient descent. Its structure consists of input, hidden, and output layers. BPNN model training consists of two sections, i.e., forward propagation of information and backpropagation of error. In forward propagation, information is passed sequentially from the input layer through the hidden layer until the output layer. The backpropagation of error is mainly used to update the weights and bias parameters in case the mean square error between the output value and the target value exceeds the target setting range. The output value of each neural node in the current layer can be calculated by Equation (5):

(5) $$y_{j}=f(\sum w_{ij}\cdot x_{i}+b_{j})(i=1,2,3,\ldots ,m;j=1,2,3,\ldots ,n)$$

where m and n indicate the neuron numbers of the current and the previous layers, respectively; $w_{ij}$ represents the connection weight between the two layers; $b_{j}$ is the threshold value of the current layer; f is an activation function; $x_{i}$ denotes the output value of the previous layer neuron; and $y_{j}$ denotes the output value of the current layer neuron.

#### 2.4.2. IGWO-SVR Model

A support vector regression (SVR) model has obvious advantages in small-sample nonlinear fitting. In sample prediction, SVR has a fast computing speed, high prediction accuracy, and fewer parameters to be adjusted. The SVR model first utilizes the kernel function to map the nonlinear data into the high-dimensional space and make the data linearly differentiable, then processes the data according to the structural risk minimization principle. However, the hyper-parameters *C* and *σ* in the SVR model should be carefully determined when applying the SVR model in practice. Consequently, we used the previously developed IGWO algorithm for hyper-parameters optimization, the implementation steps of which are described in the literature [30].

#### 2.4.3. LSTM Model

**Figure 3:**
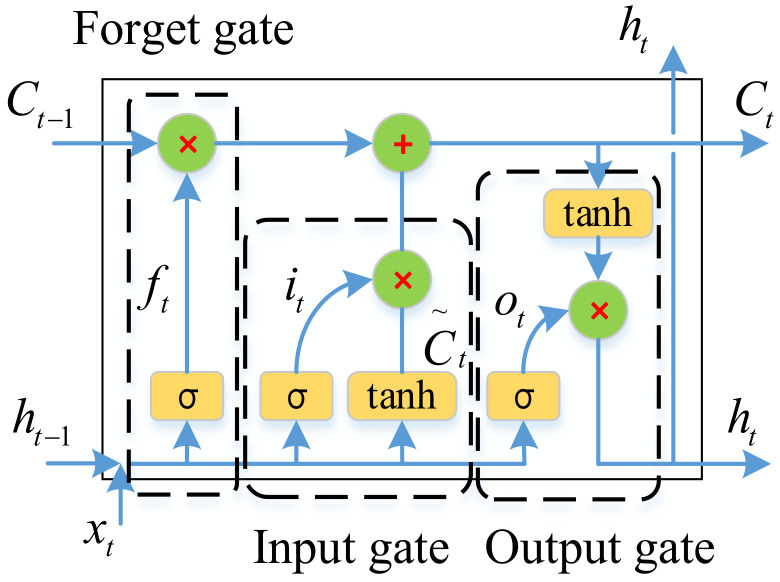
LSTM structural unit.

The LSTM network combines long-time and short-time series-related information through subtle gate control to better preserve the long-time series-related information and to control the gradient flow, which can effectively solve the gradient disappearance or explosion problem, enhancing network reliability, and is more suitable for continuous estimation of dynamic extension force. The LSTM blocks consist of the forget gate, input gate, and output gate. These units are connected in series to learn and store long-term and short-term series-related information. The standard LSTM structure [42] is shown in Figure 3. In the LSTM structure, input gates ($i_{t}$), forgetting gates ($f_{t}$), and output gates ($o_{t}$) are designed to retain or discard information, respectively.

In the LSTM structural unit, the mapping of the current input $x_{t}$ to the output $h_{t}$ at time t is calculated by the following formula [43]:

(6) $$\left\{\begin{array}{c} \begin{array}{c} f_{t}=\sigma \left(W_{f}\left[h_{t-1},x_{t}\right]+b_{f}\right)\\ i_{t}=\sigma \left(W_{i}\left[h_{t-1},x_{t}\right]+b_{i}\right)\\ {\tilde{C}}_{t}=\tanh\left(W_{C}\left[h_{t-1},x_{t}\right]+b_{C}\right) \end{array}\\ \begin{array}{c} C_{t}=f_{t}\cdot C_{t-1}+i_{t}\cdot C_{t}\\ o_{t}=\sigma \left(W_{o}\left[h_{t-1},x_{t}\right]+b_{o}\right)\\ h_{t}=o_{t}\cdot \tanh\left(C_{t}\right) \end{array} \end{array}\right.$$

where $C_{t-1}$ is the unit memory from the previous block, ${\tilde{C}}_{t}$ is the candidate information of the unit memory at the current moment, $C_{t}$ is the updated value of the cell state, and $h_{t}$ is the output value of the current block, which also serves as the input for the next time. $f_{t}$, $i_{t}$, and $o_{t}$ are the values of the forget gate, the input gate, and the output gate, respectively. $W_{*}$ and $b_{*}$ represent the corresponding weights and bias terms of the layer, respectively. In the current block, σ and tanh are two activation functions, representing the sigmoid function and the hyperbolic tangent function, respectively, through which the functions of the gating structure are implemented.

#### 2.4.4. IGWO-LSTM Model

The fitting ability and training effect of an LSTM neural network are closely related to its network parameters. Usually, the hyper-parameters of a traditional LSTM neural network depend on empirical tuning, which greatly reduces the performance of the LSTM model [42]. Therefore, we used the previously developed IGWO algorithm [40] to optimally search for LSTM hyper-parameters to construct an estimation model with appropriate hyper-parameters (e.g., the learning rate and the number of hidden layer neurons), which is both adaptable to the data of the knee extension task and displays the outstanding performance of the LSTM. In this paper, the LSTM model consisted of an input layer, a hidden layer, a fully connected layer, and a regression layer in which an Adam solver was used. In addition, the root mean square error (RMSE) was used as the fitness function.

The main idea of the proposed algorithm is to utilize the IGWO superiority capability to search the LSTM network parameters. According to the RMSE of LSTM, more suitable network parameters can be obtained. The specific steps are as follows.

Step 1: Initialize the population size and other parameters of IGWO and set the initial ranges of learning rate and hidden layer neurons and other network parameters of the LSTM.

Step 2: Determine the RMSE of the LSTM training output as the fitness function of IGWO.

Step 3: Initialize the positions of the wolf *α*, *β*, *δ*, and *ω*.

Step 4: Execute the process of encircling prey, hunting and attacking prey, and update the position of the wolf *α*, *β*, *δ*, and *ω*.

Step 5: Iterate step 4 until the iterative constraint is achieved.

Step 6: Obtain the optimal network hyper-parameters from the final α-wolf position, configure the estimation model, and finally use it for knee dynamic extension force estimation.

#### 2.4.5. Model Evaluation Indicators

To evaluate the estimation results, the estimated forces were evaluated against the observed forces based on the normalized root mean square error (NRMSE), mean absolute percentage error (MAPE), and correlation coefficient (R), which are defined as follows:

(7) $$NRMSE=\frac{\sqrt{\frac{1}{N}\sum_{i=1}^{N}{\left({\hat{y}}_{i}-y_{i}\right)}^{2}}}{y_{max}-y_{min}}$$

(8) $$MAPE=\frac{1}{N}\sum_{i=1}^{N}\left|\frac{{\hat{y}}_{i}-y_{i}}{y_{i}}\right|$$

(9) $$R=\frac{{Cov({\hat{y}}_{i},y}_{i})}{\sqrt{D({\hat{y}}_{i})}\sqrt{D(y_{i})}}$$

where ${\hat{y}}_{i}$ is the estimated value, $y_{i}$ is the actual value, $y_{max}$ and $y_{min}$ are the maximum and minimum values of the actual values, respectively, D(·) is the calculated variance, Cov(·) is the covariance, *N* is the number of samples in the test set, and the subscript i indicates the *i*-th data point. Generally speaking, the closer NRMSE and MAPE are to 0, and R to 1, the closer the estimated value of the model is to the observed value.

## 3. Results

### 3.1. Cross-Talk Analysis of Different Muscle Pairs

**Figure 4:**
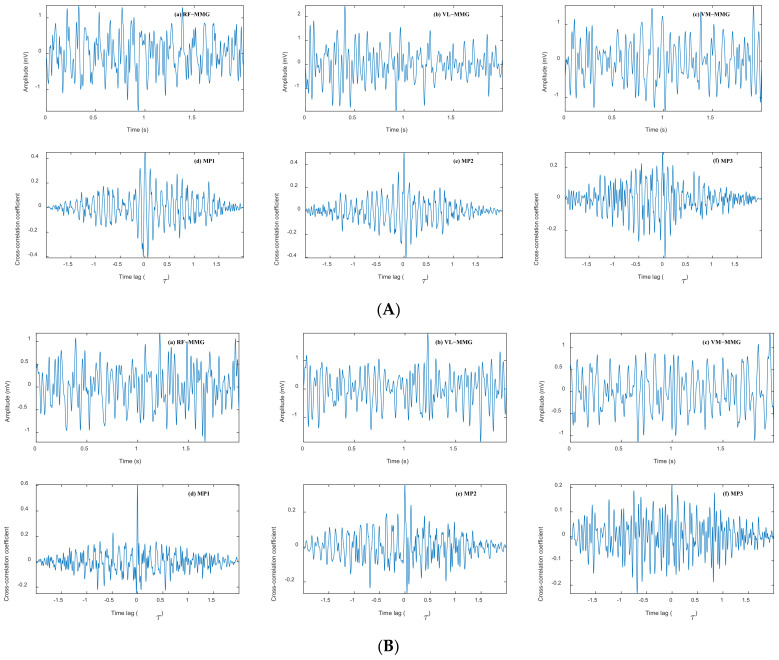
Cross-talk analysis of MMG signal segments from RF, VL, and VM. (**A**) Cross-talk analysis of MMG signal fragments at 60% MVIC. (**B**) Cross-talk analysis of MMG signal segments at DVIC (20–60% MVIC).

For cross-talk analysis, a 2 s signal segment was extracted from each muscle MMG. Additionally, cross-correlation coefficients were used to quantify cross-talk between different muscle pairs during contracting muscle. The results of the cross-correlation analysis indicate that most of the PCCC was observable at a time lag (τ) of approximately 0 s, which is consistent with the literature [44,45,46]. Figure 4 shows the MMG signals and the correlation diagrams of different muscle pairs during 60% MVIC and DVIC, respectively. As can be seen in Figure 4, MMG signals of all three muscles have cross-talk in the case of SVIC and DVIC, which poses further difficulties in fully identifying different muscle activities.

**Figure 5:**
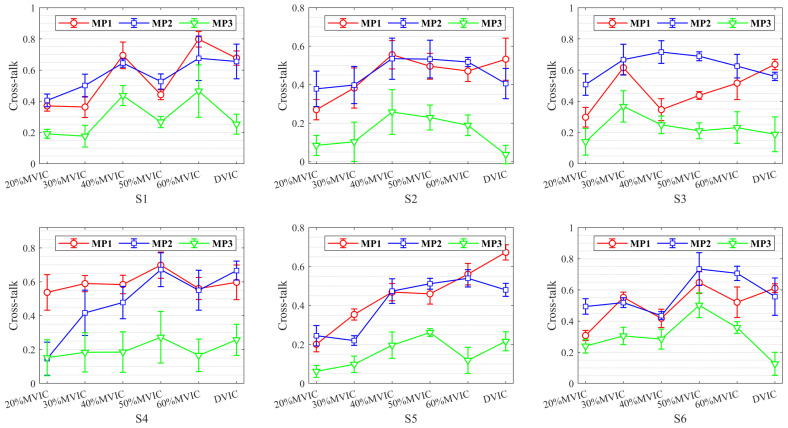
Statistical results of cross-talk analysis for different muscle pairs during SVIC and DVIC.

To investigate MMG cross-talk between different muscle pairs, the cross-talk results for six subjects at different levels of SVIC and DVIC are plotted in Figure 5. From the statistical analysis, the range of cross-talk between different muscle pairs was 0.0538–0.7978, which demonstrates that all of these adjacent muscles may contribute to the MMG signals, i.e., all muscles are contaminated by cross-talk.

As can be seen in Figure 5, there is an overall non-significant trend of increased cross-talk for all muscle pairs during the muscle force from 20% MVIC to 60% MVIC. In addition, there is a significant difference in the magnitude of cross-talk between the MP1, MP2, and MP3 muscle pairs. In almost all isometric muscle contractions, cross-talk between adjacent muscle pairs (MP1 and MP2) is greater than the cross-talk between non-adjacent muscle pairs (MP3). In particular, during DVIC, MP1 cross-talk is greater than MP2 for all subjects, while MP2 was greater than MP3, except for subject S4, whose weight was far more than normal standards. The cross-talk between MP1 and MP2 was much higher than MP3 in all subjects, indicating that RF contains a large amount of muscle information from VL and VM. These also further demonstrate that muscle force estimation using MMG signals obtained from three muscles or RF may lead to unpredictable inaccuracies, which implies that muscle force estimation using MMG signals obtained from VL or VM muscles may perhaps improve estimation accuracy.

### 3.2. Knee Dynamic Extension Force Estimation with Different Muscle Feature Combinations

Usually, a single feature or a small number of features can lead to data loss; too many features can also lead to data redundancy and possibly even dimensional disaster. Therefore, in this paper, effective features highly related to knee extension force were extracted from RF, VL, and VM, respectively, using GRA to reflect muscle activity. The different feature combinations extracted from MMG signals using GRA in this paper are defined as follows: the features extracted from RF are defined as F1; the features extracted from VL are defined as F2; the features extracted from VM are defined as F3; the features extracted from RF and VL are defined as F4; the features extracted from RF and VM are defined as F5; the features extracted from VL and VM are defined as F6; and the features extracted from RF, VL, and VM are defined as F7. To test the effect of knee dynamic extension force estimation with different feature combinations, we used fixed hyper-parameters of the LSTM model with the number of hidden layer neurons of 100, the initial learning rate of 0.005, and the max. epochs of 250. 

In addition, we selected the first 90% of the data sequences as the training sample and the last 10% of the data sequences as the test sample. To suppress data that is too large or too small, the data were standardized using the Z-score method. From the cross-talk analysis and the quadriceps anatomy, it can be seen that estimating knee dynamic extension force from all features obtained from RF, VL, and VM may lead to poor estimation results. Therefore, we can further hypothesize that knee dynamic extension force estimation by a single muscle with a small level of cross-talk can improve accuracy and validity.

**Figure 6:**
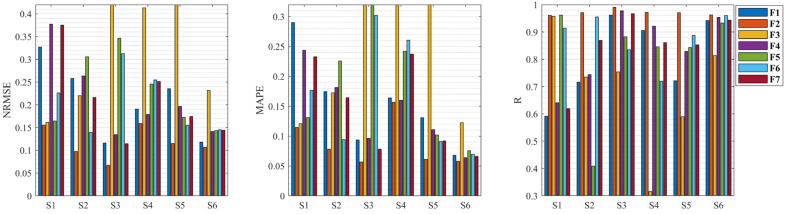
Estimated results of knee dynamic extension force during isometric contraction with different muscle feature combinations for six subjects.

To verify the above hypothesis, the results of knee dynamic extension force estimation for six subjects with different feature combinations is illustrated in Figure 6 and Table 1, respectively. Figure 6 shows the NRMSE, MAPE, and R of the LSTM model on the test sample under different feature combinations for six subjects. From the estimated results for six subjects with different feature combinations in Figure 6, it can be seen that each subject’s feature combination F2 presents optimal results in comparison to the other feature combinations.

The mean estimation results of different feature combinations in Table 1 show that the feature combination F2 provides the optimal results, with NRMSE of 0.1167, MAPE of 0.0875, and R of 0.9714, and with a smaller standard deviation, i.e., the estimated results of the feature combination F2 are more stable than those of other feature combinations. This also demonstrates that MMG signals from VL for knee dynamic extension force estimation are superior to MMG signals from the other two muscles, which may be due to the fact that the MMG from VL has less cross-talk and describes the muscle activity more directly during knee extension. As a result, these estimation results are in general agreement with the results of the cross-talk analysis, indicating that the muscle with larger cross-talk and multiple muscles has a non-negligible effect on knee extension force estimation, while a single muscle with less cross-talk can provide a more accurate knee extension force estimation, further confirming the validity of our hypothesis.

### 3.3. Applying the IGWO-LSTM Model to Estimate Knee Dynamic Extension Force

To further improve the accuracy of knee dynamic extension force estimation, we used the IGWO algorithm to optimize the LSTM hyper-parameters. In this paper, the number of hidden layer neurons was set in the range [50, 400], the initial learning rate was set in the range [0.001, 0.01], the max. epochs was set to 300 to prevent under-fitting, and the other parameters were set in the same way as the LSTM model with the fixed hyper-parameters. In addition, the population size and the maximum number of iterations in the IGWO algorithm were set to 10 and 20, respectively. 

**Figure 7:**
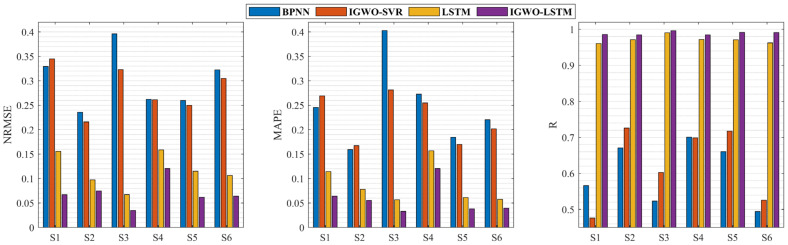
Comparison of the performance of different models in knee dynamic extension force estimation for six subjects.

To verify the validity of the proposed IGWO-LSTM model for knee dynamic extension force estimation, a comparison was carried out using BPNN, IGWO-SVR, and LSTM. The results of different estimation models for the same dataset are shown in Figure 7 and Table 2. It can be seen that among different knee dynamic extension force estimation models for six subjects the IGWO-LSTM model achieves the best results with NRMSE of 0.0704, MAPE of 0.0583, and R of 0.9891. Additionally, the estimation results of the LSTM with fixed hyper-parameters reveal poorer and less stable results, especially in the NRMSE and MAPE performance indicators. As a result, it is shown that the proposed IGWO-LSTM model is utilized to not only adaptively configure the LSTM hyper-parameters, but also to obtain better estimation results based on the knee dynamic extension task data.

**Figure 8:**
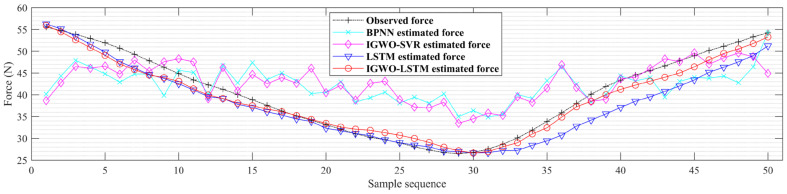
Knee dynamic extension force estimation of different estimation models.

Figure 8 shows the estimation results of different estimation models for subject S3 in the test sample, with the black line showing the observed force sequence, the red line showing the estimated sequence from the IGWO-LSTM model, and the blue line showing the estimated sequence from the LSTM model. In addition, the estimated sequences from BPNN and IGWO-SVR are also plotted in Figure 8. The closer the estimated sequence is to the black line, the higher the accuracy of the model’s estimation. As can be seen in Figure 8, the estimated sequence of the BPNN and IGWO-SVR models are farther away from the observed sequence, indicating poorer estimation results, while the estimated sequence of the IGWO-LSTM model is closest to the observed sequence. These results indicate that the IGWO-LSTM model is far more accurate than any other model, and further demonstrate that the proposed model is more suitable for high-precision knee dynamic extension force estimation.

## 4. Discussion

In most voluntary muscle contractions, the muscle fibers are activated, and the mechanical activity of the motor unit is superimposed non-linearly to form MMG signals. MMG signals generated during voluntary muscle contraction are much more complex than those recorded during neuromuscular electrical stimulation (NMES). The simultaneous activation of different muscles facilitates a single-limb task. However, during knee dynamic extension in all subjects, we observed cross-talk in MMG signals from all three muscles of the quadriceps. Consequently, all of these muscles may contribute to the MMG signals during knee extension.

The most straightforward way to assess cross-talk is to detect signals from other muscles when selectively activating one muscle. In practice, however, it is often difficult to selectively activate a single muscle, as reflex arcs can lead to other muscles being activated, resulting in inaccurate estimates of cross-talk conduction. However, there is a significant advantage in assessing cross-talk using the CCC method, which is not very complex and only requires MMG signals from the muscles. The cross-correlation coefficients are used to quantify cross-talk as it is more readily available, and to obtain the proportion of common signals between two muscles without knowing any information about uncontaminated signals. Despite criticisms of the use of CCC in past studies [10], it is currently the most powerful method for quantifying cross-talk. This assessment supports the studies of Islam et al. [41] and Talib et al. [16], showing a strong positive correlation between cross-talk amplitude and isometric contraction levels. These studies have all indicated that there is a certain degree of cross-talk between adjacent muscles. However, this paper indicates that the cross-talk magnitude between MP1, MP2, and MP3 muscle pairs differs significantly, which has some similarities to the above literature results, and that cross-talk between adjacent muscles (MP1, MP2) is greater than that between non-adjacent muscles (MP3) during almost all isometric contractions. This may be based on the fact that since non-adjacent muscles contain more connective and adipose tissue (e.g., skin, bone, subcutaneous fat) than adjacent muscles, it may attenuate the signal strength during muscle contraction, thereby reducing the cross-talk amplitude between non-adjacent muscles [46]. In addition, as can be seen from the anatomy in Figure 1, RF is very near to VL, VM; VL is not directly adjacent to VM, further confirming the above observation.

In the experiments, it was assumed that using a single muscle to obtain MMG signals for estimating knee dynamic extension force may improve the estimation accuracy. Among the different feature combinations, we observed that the estimation results using MMG signals obtained from RF and VM are not satisfactory, whereas the estimation results using MMG signals obtained from VL were very accurate. The result may be explained by the fact that MMG signals obtained from the RF are more contaminated by the VL and VM, and that VM is in close physiological contact with other surrounding muscles resulting in more complex MMG signals. Compared to RF and VM, MMG signals obtained from VL are less contaminated by other muscles and provides a visual and accurate representation of extension force. It also further demonstrates that knee extension involves various muscles, some of which primarily assist and maintain force, whereas the activity information of some muscles intuitively represents subtle fluctuations of knee extension force.

In addition, compared with BPNN, IGWO-SVR, and LSTM, the proposed IGWO-LSTM model can obtain superior accuracy in estimating the knee dynamic extension force. We observed that knee dynamic extension force estimation with BPNN yields extremely unstable results with large deviations between two adjacent results, as well as the poor stabilization results achieved by the IGWO-SVR. Although BPNN and IGWO-SVR achieved excellent results in knee static extension force estimation, they performed poorly in knee dynamic extension force estimation, which could be due to two main reasons: firstly, MMG signals are more complex during knee dynamic force compared to knee static force; secondly, the current muscle contraction is associated with the pre-muscle contraction.

The primary aim of this study is two-fold: (1) to explore the feasibility of selecting a single muscle from a cross-talk analysis to estimate knee dynamic extension force, and (2) to design an effective estimation model. Through experimental comparison, the proposed IGWO-LSTM estimation model is optimal and can estimate knee dynamic extension force with higher accuracy, and the obtained results are better than the estimation results in the literature [18,28,29,47]. Moreover, a sliding overlap length of 50 ms was used for feature extraction during signal processing, which provided good real-time performance.

However, the present study includes some limitations that will be addressed in future research. Firstly, this study ignores the fact that muscle fatigue may produce muscle stiffness. Secondly, due to the introduction of the IGWO algorithm, it is necessary to train the LSTM network several times, which will consume more extra computational cost and time accordingly. Once suitable hyper-parameters are obtained after the optimization of the IGWO algorithm, it will bring promising estimation effects for estimating knee dynamic extension force. Finally, the experiments are limited to knee extensors during isometric muscle contraction; however, in many cases the knee joint often involves complex reciprocating movements, requiring consideration of a wider range of muscle groups.

## 5. Conclusions

In conclusion, this paper presents the results of a study on knee dynamic extension force estimation utilizing cross-talk analysis and IGWO-LSTM. In contrast to prior research on MMG signals for muscle force estimation, our proposed scheme can be used to accurately estimate knee dynamic extension force. Firstly, owing to the existence of cross-talk between different muscles, the hypothesis is given that employing MMG signals from a single muscle with less cross-talk can increase the accuracy of knee dynamic extension force estimation. Through cross-talk analysis and different feature combination experiments, the results demonstrate that this hypothesis is correct, providing a foundation for accurate knee dynamic extension estimation. Then, a novel estimation model (IGWO-LSTM) is proposed to further improve the performance of knee dynamic extension force estimation. The results show that the proposed IGWO-LSTM achieves the optimal performance indicators with NRMSE of 0.0704, MAPE of 0.0583, and R of 0.9891 compared to other state-of-the-art models. Therefore, this study has great potential for application in rehabilitation and assistive devices. Additionally, to develop more natural and flexible device control, it is necessary to continue to investigate the limitations mentioned in Section 4 in the future.

## Figures and Tables

**Table 1:** Mean results (mean ± std) of knee dynamic extension force estimation for six subjects with different feature combinations.

Feature Combination	NRMSE	MAPE	R
F1	0.2076 ± 0.0827	0.1534 ± 0.0783	0.8066 ± 0.1507
F2	0.1167 ± 0.0352	0.0875 ± 0.0404	0.9714 ± 0.0106
F3	0.3794 ± 0.2080	0.3158 ± 0.2134	0.6943 ± 0.2202
F4	0.2151 ± 0.0918	0.1427 ± 0.0654	0.8443 ± 0.1320
F5	0.2295 ± 0.0830	0.1884 ± 0.1052	0.8126 ± 0.2031
F6	0.2055 ± 0.0705	0.1656 ± 0.0978	0.8788 ± 0.0905
F7	0.2126 ± 0.0934	0.1450 ± 0.0776	0.8522 ± 0.1233

**Table 2:** Mean results (mean ± std of different estimation models for six subjects.

	BP	IGWO-SVR	LSTM	IGWO-LSTM
NRMSE	0.3008 ± 0.0596	0.2833 ± 0.0490	0.1168 ± 0.0352	0.0704 ± 0.0280
MAPE	0.2476 ± 0.0863	0.2241 ± 0.0508	0.0875 ± 0.0404	0.0583 ± 0.0326
R	0.6028 ± 0.0860	0.6244 ± 0.1065	0.9714 ± 0.0106	0.9891 ± 0.0048

## Data Availability

The data that support the findings of this study are available from the corresponding author upon reasonable request.
